# Improved Model of Proton Pump Crystal Structure Obtained by Interactive Molecular Dynamics Flexible Fitting Expands the Mechanistic Model for Proton Translocation in P-Type ATPases

**DOI:** 10.3389/fphys.2017.00202

**Published:** 2017-04-11

**Authors:** Dorota Focht, Tristan I. Croll, Bjorn P. Pedersen, Poul Nissen

**Affiliations:** ^1^Department of Molecular Biology and Genetics, Aarhus UniversityAarhus, Denmark; ^2^DANDRITE, Nordic-EMBL Partnership for Molecular Medicine, Aarhus UniversityAarhus, Denmark; ^3^PUMPkin, Danish National Research Foundation, Aarhus UniversityAarhus, Denmark; ^4^Institute of Health Biomedical Innovation, Queensland University of TechnologyBrisbane, QLD, Australia; ^5^Aarhus Institute of Advanced Studies, Aarhus UniversityAarhus, Denmark

**Keywords:** P-type ATPases, plasma membrane proton pump, proton gradient, *Arabidopsis thaliana* AHA2, molecular dynamics, iMDFF, crystallography, membrane transport

## Abstract

The plasma membrane H^+^-ATPase is a proton pump of the P-type ATPase family and essential in plants and fungi. It extrudes protons to regulate pH and maintains a strong proton-motive force that energizes e.g., secondary uptake of nutrients. The only crystal structure of a H^+^-ATPase (AHA2 from *Arabidopsis thaliana*) was reported in 2007. Here, we present an improved atomic model of AHA2, obtained by a combination of model rebuilding through interactive molecular dynamics flexible fitting (iMDFF) and structural refinement based on the original data, but using up-to-date refinement methods. More detailed map features prompted local corrections of the transmembrane domain, in particular rearrangement of transmembrane helices 7 and 8, and the cytoplasmic N- and P-domains, and the new model shows improved overall quality and reliability scores. The AHA2 structure shows similarity to the Ca^2+^-ATPase E1 state, and provides a valuable starting point model for structural and functional analysis of proton transport mechanism of P-type H^+^-ATPases. Specifically, Asp684 protonation associated with phosphorylation and occlusion of the E1P state may result from hydrogen bond interaction with Asn106. A subsequent deprotonation associated with extracellular release in the E2P state may result from an internal salt bridge formation to an Arg655 residue, which in the present E1 state is stabilized in a solvated pocket. A release mechanism based on an in-built counter-cation was also later proposed for Zn^2+^-ATPase, for which structures have been determined in Zn^2+^ released E2P-like states with the salt bridge interaction formed.

## Introduction

The *Arabidopsis thaliana* plasma membrane H^+^-ATPase 2 (AHA2) is a member of the P_III_-subtype of the P-type ATPase superfamily. It pumps protons out of the cell to maintain a steep electrochemical H^+^ gradient and potential across the plasma membrane (Serrano et al., 1986; Blatt et al., 1987). The H^+^-ATPases are of fundamental importance in plants and fungi, as well as several prokaryotes (Pedersen et al., 2014). They maintain a membrane potential at around −150 mV in plants, even down to −300 mV in fungi, control intracellular H^+^ homeostasis and extracellular acidification, and potentiate secondary transporters involved in e.g., nutrient uptake (Briskin, 1990).

P_III_-type H^+^-ATPases are substantially different in sequence and function to the P_II_-subtype ATPases such as the gastric H^+^/K^+^-ATPases in animals. A total of 11 isoforms of H^+^-ATPases have been identified in *A. thaliana* (Harper et al., 1994), of which AHA1 and AHA2 are the most abundantly expressed in the plasma membrane. AHA1 and AHA2 can compensate for each other, while the deletion of both genes is lethal (Haruta et al., 2010). *Neurospora crassa* has only one plasma membrane H^+^-ATPase (PMA), which is essential for cell growth. In *Saccharomyces cerevisiae* two isoforms (PMA1 and PMA2) are found, of which PMA1 is constitutively expressed and essential (Serrano et al., 1986), but AHA2 can compensate a PMA1 knockout (Palmgren and Christensen, 1993). Due to the important role of plasma membrane H^+^-ATPases for cellular life in plants and fungi, and their significant differences to human pumps, they represent attractive targets for anti-fungal and herbicidal strategies (Schubert and Peura, 2008; Yatime et al., 2009).

**Figure 1:**
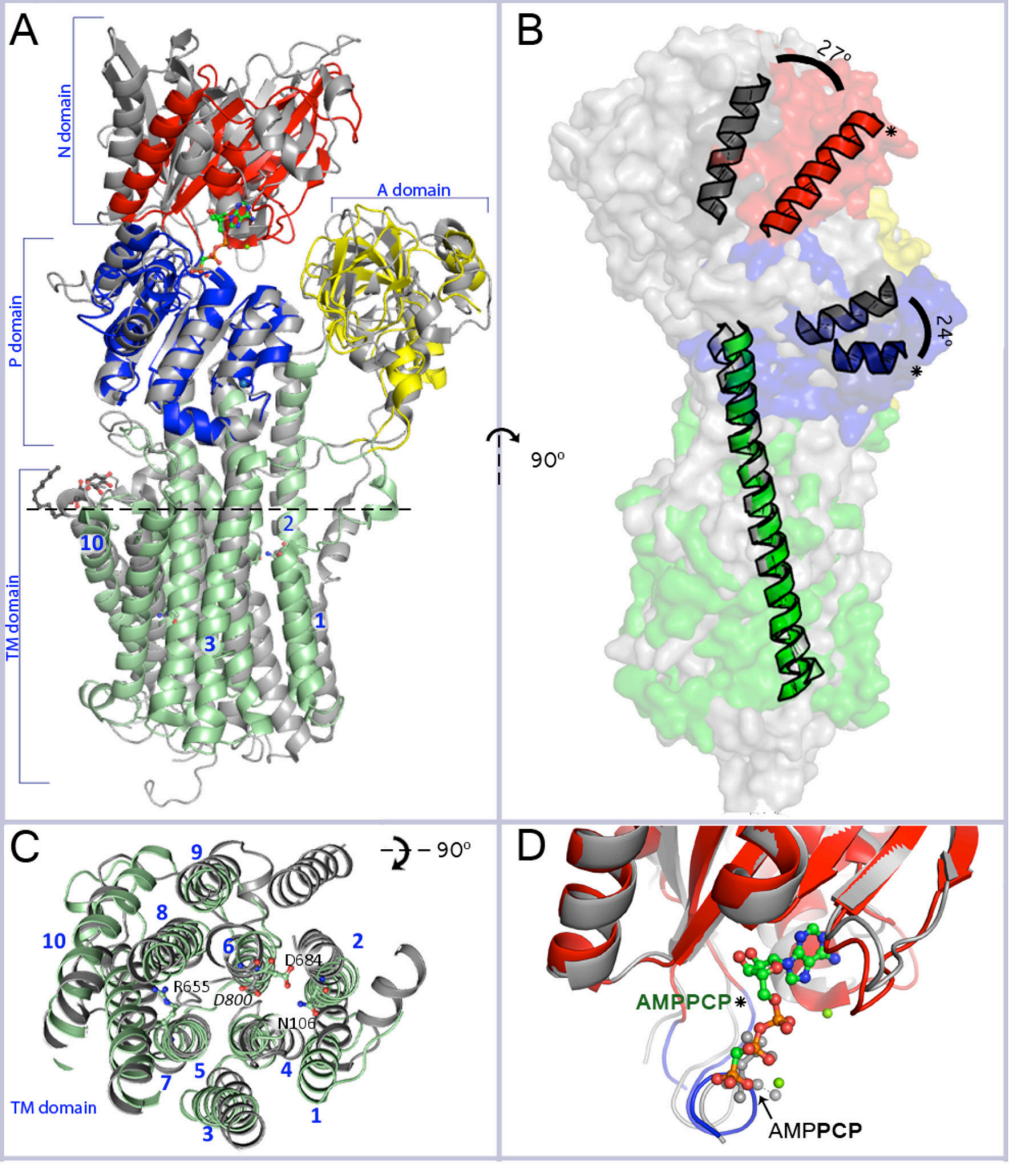
**Overall resemblance of AHA2-AMPPCP to the SERCA-SLN complex. (A)** Superposition of the revised structure of AHA2-AMPPCP (domains colored as indicated) and the SERCA-SLN complex (gray; pdb id: 4H1W) both representing E1 state. The structures are superimposed on Cα position of the TM or P domains only. Mg^2+^ and K^+^ ions are shown by green and purple-blue spheres, respectively. The spatial organization of the cytoplasmic domains is similar in both E1 state structures. Two loops connecting the A-domain with TM1 and TM3, respectively, align well in AHA2 and SERCA, although for the latter TM1 is longer and kinks into a short helix parallel to the membrane. The TM3 connector forms a longer helix in the A-domain of SERCA compared to AHA2. The N-domain is moved slightly closer toward the A-domain in the SERCA structure. The adenine base part of the AMPPCP molecule is coordinated by side chains of Asp372 and Asp375 and the backbone oxygen of Ser457. The major differences between the P-domains are visible in a region between Pro533^AHA2^ to Asp559^AHA2^, which covers one loop and two short helixes. **(B)** Side view of the aligned pumps (superimposed on TM2–TM10 domains, r.m.s.d 3.07 Å for 159 Cα atoms) showing a tilt of the cytoplasmic headpiece of AHA2 (in colors) relatively to SERCA (gray). The N- and P-domain headpiece of AHA2 is tilted by ~25° (measured between Cα of Ser436^AHA2^ and Thr538^SERCA^ for the N-domain and between Cα of Pro550^AHA2^ and Pro662^SERCA^ for the P-domain, using Cα of Lys625^AHA2^ in TM5 in both cases as an apex). Asterisks mark helices from the P- and N-domain of AHA2. **(C)** Alignment of the transmembrane region. Top view from the plane marked by dashed line at segment A. Catalytically important residues of AHA2 and conserved Asp800 of SERCA are labeled. **(D)** Superposition of the N-domain of AHA2 and SERCA-SLN (r.m.s.d 0.97 Å for 123 atoms superimposed Cα atoms), both in E1 states and showing overlapping binding of β-γ phosphates of AMPPCP. The AMPCPP molecule represents the revised AHA2 model.

Overall, the P_III_-type plasma membrane H^+^-ATPases share a similar fold with the P_II_ subfamily including Na^+^/K^+^-ATPases, H^+^/K^+^-ATPases, and Ca^2+^-ATPases present in animal cells (Bublitz et al., 2011). Structural organization of AHA2 encompasses a cytoplasmic headpiece formed by three domains; the nucleotide (N) binding domain, the phosphorylation (P) domain, and the actuator (A) domain, as well as a membrane domain composed of 10 transmembrane segments harboring the proton binding site and the translocation pathway (Figure 1A; Pedersen et al., 2007). Additionally and similar to other members of the P_III_ subfamily (Mandala and Slayman, 1989; Portillo et al., 1989), AHA2 has N- and C-terminal extensions involved in auto-regulatory functions (Ekberg et al., 2010a), the latter being considerably longer and referred to as the R-domain. The first and so far only direct structure determination of a H^+^-ATPase was obtained of AHA2 in 2007 from highly anisotropic, low-resolution crystallographic data (Pedersen et al., 2007). Model building and refinement with difficult data of this kind remains very challenging even today, and the 2007 model reflected this although it clearly expanded our knowledge on the spatial organization and architecture of AHA2 and afforded models on the transport mechanism.

Here, we have made use of new model-building and refinement tools to present a new, re-refined atomic model of AHA2, which can be employed for more accurate structural comparisons and models of transport. Rebuilding and re-refinement employed the new interactive molecular dynamics flexible fitting (iMDFF) approach (Croll and Andersen, 2016) in combination with phenix.refine (Adams et al., 2010) and resulted in a significantly improved model with new features emerging in the electron density maps. The revised model as well as the body of published data since 2007 invites a reiteration of the structure/function relationship, and it allows also tentative studies by molecular dynamics simulations in a lipid bilayer environment.

## Materials and methods

### Rebuilding and refinement

Revision of the AHA2 model representing two copies (chain A and B) in the asymmetric unit of a P2_1_2_1_2_1_ crystal form was initiated from the coordinates of Protein Data Bank entry 3B8C, via multiple rounds of rebuilding in the iMDFF environment (Croll et al., 2016), interspersed with refinement in PHENIX. Parameters for modeling of AMPPCP and DDM molecules in iMDFF were obtained using the CGENFF server (Vanommeslaeghe and MacKerell, 2012; Vanommeslaeghe et al., 2012), and for refinement in phenix.refine using phenix.elbow. The first few rounds of rebuilding/refinement were carried out using the elliptically truncated data used for a major part of the refinement/rebuilding cycles in the original analysis, but we later observed that using data reprocessed without elliptical truncation improved the stability of refinements substantially, and this was used from then on. As previously observed (Croll and Andersen, 2016), we found that the use of a TLS-only B-factor model (i.e., no individual B-factor refinement) improved the interpretability of maps, particularly during initial rounds. Worth noting, the combination of this simplified B-factor model and the strict handling of non-bonded interactions in the iMDFF environment led to an initial dramatic increase in *R*_*free*_ from 0.366 to ~0.42 as regions making unfavorable interactions were pushed out of density, and it took several rounds of rebuilding and refinement before the R-factors started to drop below those of the original model.

In each round of rebuilding the entire structure was inspected end-to-end via localized iMDFF simulations, with a typical simulation containing 100 contiguous residues as well as the surrounding shell of residues approaching within 5 Å of these. Secondary structure was visualized by a standard cartoon overlay over the backbone atoms and updated every 50 simulation steps, while the status of residues on the Ramachandran plot was mapped to the color of the Cα atoms and updated every five steps. Small corrections such as side chain rotamers or backbone geometry were fixed on-the-fly by either simply tugging on atoms with a haptic interface or applying scripted forces to guide side chain dihedrals toward library target values. Where more substantial rearrangements were deemed necessary (e.g., rearrangement of a flexible loop or shifting the register of a secondary structure element), more localized simulations were run both to limit impact on surroundings and to provide sufficient simulation performance for interactivity. Register shifts were aided by scripted forces, applying position targets to Cα atoms to make the shift and dihedral targets to the backbone φ and ψ angles to maintain secondary structure geometry. All simulations were performed under generalized Born implicit solvent conditions. During most interactive remodeling the simulation temperature was maintained at 100 K. Prior to writing coordinates for crystallographic refinement the entire structure was settled by reducing the temperature from 100 to 0 K in 20 K increments over 10–20 ps of simulation time.

Refinement in PHENIX was typically via the following protocol. A TLS-only B-factor model was first refined in 4–8 rounds with coordinates fixed. This was followed by a further 5–10 rounds of coordinate and TLS refinement with torsion-angle restraints using the input model as a reference. Reference model restraints were then released, and a further 5–10 rounds run with torsion-angle NCS restraints. In the last few rebuilding/refinement rounds, a final refinement step was added to refine individual B-factors.

### Molecular dynamics simulation

Equilibrium simulations in an explicit membrane/water environment were carried out in NAMD (Phillips et al., 2005) using the CHARMM36 force field (Huang and MacKerell, 2013) in the NPT ensemble with periodic boundary conditions at a rate of 1 fs per time step. Van der Waals interactions were calculated every two time steps and electrostatics every four time steps. The temperature was set to 298 K and pressure to 1 atmosphere. A POPC lipid bilayer was built in VMD (Humphrey et al., 1996), and a single monomer (chain B) of the refined coordinates embedded in it. AMPPCP was handled as ATP. Phospholipid molecules with atoms overlapping protein atoms were deleted, and the resulting construct was solvated in a box of 46,503 TIP3P water molecules and neutralized with 0.15 M KCl. The system was energy minimized for 2,000 steps, and equilibrated for 1 ns with all protein atoms and the ATP fixed in space to dehydrate the membrane and allow settling of lipid around the protein. The simulation was continued for a further 3 ns with side chain atoms and ATP free to move, but with backbone atoms held by harmonic restraints. Finally, the simulation was run unrestrained for 30 ns.

### Summary of key changes to the AHA2 model

At a cursory glance, the structure appears similar, but the result is a noticeable improvement of quality scores over the original model (Pedersen et al., 2007), exemplified by the MolProbity score being reduced from 4.52 to 1.67 (Chen et al., 2010). Secondary structure is improved throughout, with 94% of residues now falling in the most favored regions of the Ramachandran plot. *R*_*free*_ is reduced from 36.6% (with anisotropic truncation) to 32.8% (without anisotropic truncation). Although still a quite high value we ascribe it to a large part to the anisotropic nature of the low resolution data set. All-atom root mean square deviation (r.m.s.d.) between the original and revised structure [using “align” command in PyMOL (Schrödinger)] is 1.86 Å (4648 atoms), while for Cα atoms the r.m.s.d. is 1.32 Å (569 atoms).

**Figure 2:**
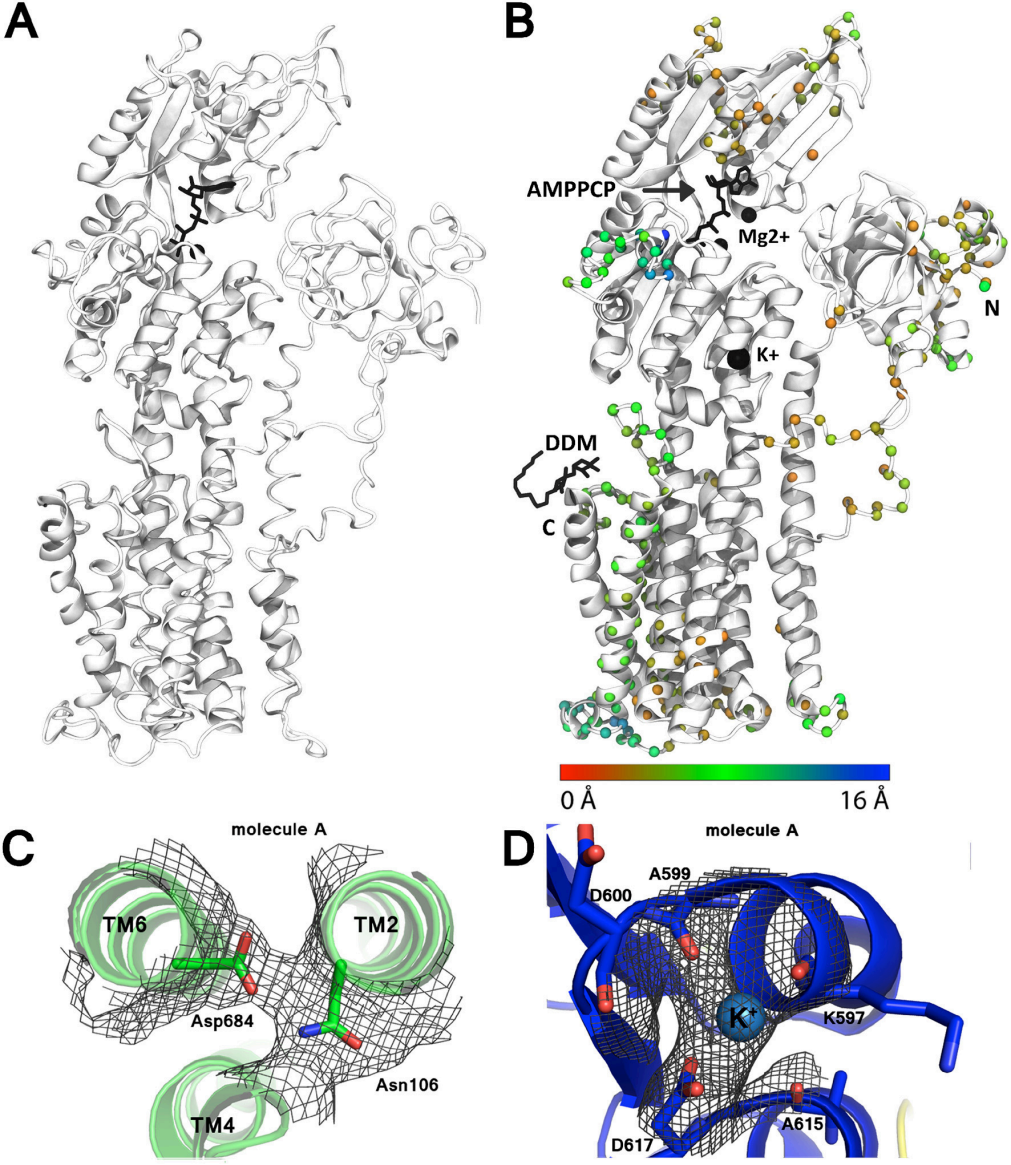
**Improvement of the AHA2 model. (A)** The initial AHA2 model (pdb id: 3B8C). **(B)** Final, revised model after re-refinement (pdb id: 5KSD). Spheres mark changes in the position of the Cα atoms between the two models (coloring according to the legend below). N and C-termini are indicated; DDM indicates bound n-Dodecyl β-D-maltoside. **(C)** Electron density map contoured at 1.0 r.m.s.d for the Asn106-Asp684 pair. **(D)** Electron density map contoured at 2.0 r.m.s.d. for the K^+^ ion binding site.

Perhaps the most important revision to the model is the rearrangement of transmembrane helices 7 and 8, which have been *N*-terminally shifted by 4 and 3 residues respectively, i.e., ~1-turn (Figure 2). Modeling of these helices was originally complicated by poor definition of the intervening loop, no significant sequence identity to e.g., SERCA, and threading of the loop after helix 8 through a tube of strong density, which however now appears more consistent with the disaccharide head-group of a DDM detergent molecule not identified in the original maps.

For the most part density associated with the cytoplasmic domains was substantially weaker than in the transmembrane region. Single-residue register shifts were also applied to parts of the N domain (residues 414–424 and 344–364), significantly changing the adenosine pocket of the ATP binding site. We were also able to resolve a conserved K^+^ binding site in the vicinity of Asp617 (Ekberg et al., 2010b) of the P-domain, and significant rearrangements were made to *N*-terminal residues 12–50 and residues 529–548 on the backside of the P domain.

## Structural and functional analysis of AHA2

### P-type ATPase catalytic cycle

**Figure 3:**
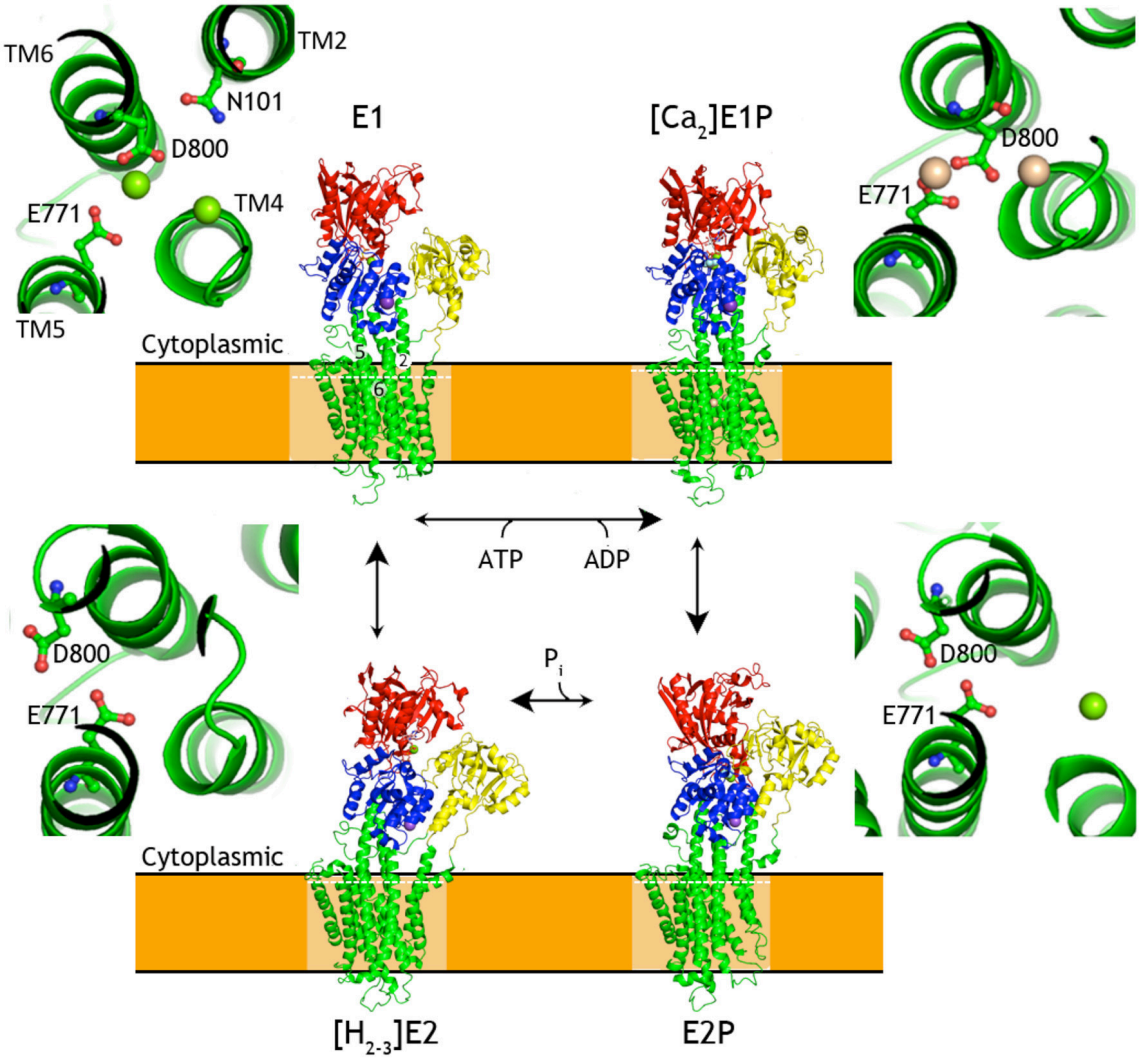
**Overview of the P-type ATPases cycle**. Schematic presentation of structural changes occurring during the catalytic cycle of P-type ATPases, represented by SERCA structures. Insets show changes in relative orientation of the residues involved in formation of SERCA ion binding throughout the transport cycle. White dashed lines mark the planes corresponding to the zoomed cross-sections. Cytoplasmic domains are colored as P-domain in blue, N-domain in red, A-domain in yellow. The transmembrane domain is shown in green. Ligands and ions are shown as spheres (K^+^ in purple-blue, Na^+^ in purple, Ca^2+^ in wheat, Mg^2+^ in green) and ball-and-sticks representation for nucleotides and metallofluorides. SERCA1a [structures 4H1W (Winther et al., 2013), 1T5T (Sørensen et al., 2004), 3B9B (Olesen et al., 2007), 2C8K (Jensen et al., 2006)]. The structures were aligned by their TM domains on TM7-TM10.

Conformational changes of P-type ATPases, shuttling between E1 and E2 states through E1P and E2P phosphoenzyme intermediates, are described by the Post-Albers cycle (Figure 3; Albers et al., 1963; Post and Sen, 1965; Pedersen and Carafoli, 1987). The resulting alternating access transport mechanism, in which the binding site of the protein is accessible only from one site of the membrane at any moment, is a prerequisite for active transport (Jardetzky, 1966). From a structural point of view the cycle is particularly well described for SERCA with overall characteristics also expected to be representative for P_III_-ATPases.

In the E1 state of a P-type ATPase the cytoplasmic substrate (H^+^ in case of AHA2, Ca^2+^ in case of SERCA) binds with high affinity at membraneous site(s). This in turn triggers rearrangement of the M1-M4 helices for robust occlusion, as well as of the A-domain to stabilize a tight approach of the N-domain with bound ATP to the P-domain with a Mg^2+^ binding site that coordinates the γ-phosphate of ATP at a conserved Asp-Lys-Thr-Gly motif (DKTG). Presence of the Mg^2+^ ion and the Lys side chain of DKTG compensate opposing negative charges of the γ-phosphate and the Asp side chain (Asp329 in AHA2) and promote phosphoryl transfer (Sørensen et al., 2004; Toyoshima et al., 2004) resulting in formation of the fully occluded, covalent aspartyl-phosphoanhydride intermediate (E1P).

The γ-phosphate transfer breaks the ATP mediated linkage between the P- and N-domain and allows the transition of the pump to the outward-facing E2P state observed for SERCA, Na^+^, K^+^-ATPase, and P1B-ATPases (Olesen et al., 2007; Yatime et al., 2011; Andersson et al., 2014; Wang et al., 2014), where the transported ions are extruded from low-affinity sites. The transition is caused by withdrawal of the N-domain that yields space for the A-domain to interact closely with the phosphorylated P-domain. In doing so, the A-domain rotates ~120° and places a conserved TGES motif in close interaction with the phosphorylated DKTG motif of the P-domain, which overall rotates by ~15° relative to the membrane. The A-domain movement affects the configuration of transmembrane helices and the ion binding site(s) in the transmembrane domain, resulting in the opening of the exit pathway toward the extracellular site of the membrane.

Counterion interactions at the membraneous site stimulate reclosure of the extracellular access pathway. The occlusion induces a small rotation of the A-domain, which engages the Glu side chain of the TGES motif to catalyze the hydrolysis of the phosphorylated Asp side chain. Subsequent release of the liberated phosphate promotes further rotation of the A-domain, away from the P domain. The pump can now return to the cytoplasmically oriented E1 state along with counterion release to the cytoplasm for those P-type ATPases performing this transport also.

### Overall structure of the AHA2 E1-AMPPCP complex

The structure of AHA2 obtained at pH 6.0 and with the ATP analog AMPPCP shows an overall typical E1 arrangement of domains, and an upright angle of the cytoplasmic headpiece relative to the membrane domain (Møller et al., 2010), although not identical to SERCA. Importantly, the E1 state observed in the structure of SERCA with sarcolipin (SLN) reported in 2013 (Toyoshima et al., 2013; Winther et al., 2013) appears to be functionally equivalent to the AHA2 state, both stabilized by AMPPCP and with the three cytoplasmic domains approaching a closed E1P conformation (Figure 1). Superpositioning of the highly conserved P-domain (r.m.s.d = 0.94 Å on Cα atoms) reveals an overlapping position of the N domain (Figure 1A), which is however noticeably smaller in AHA2 and rotated by ~25°, when the structures are aligned by the transmembrane domains (Figure 1B).

**Figure 4:**
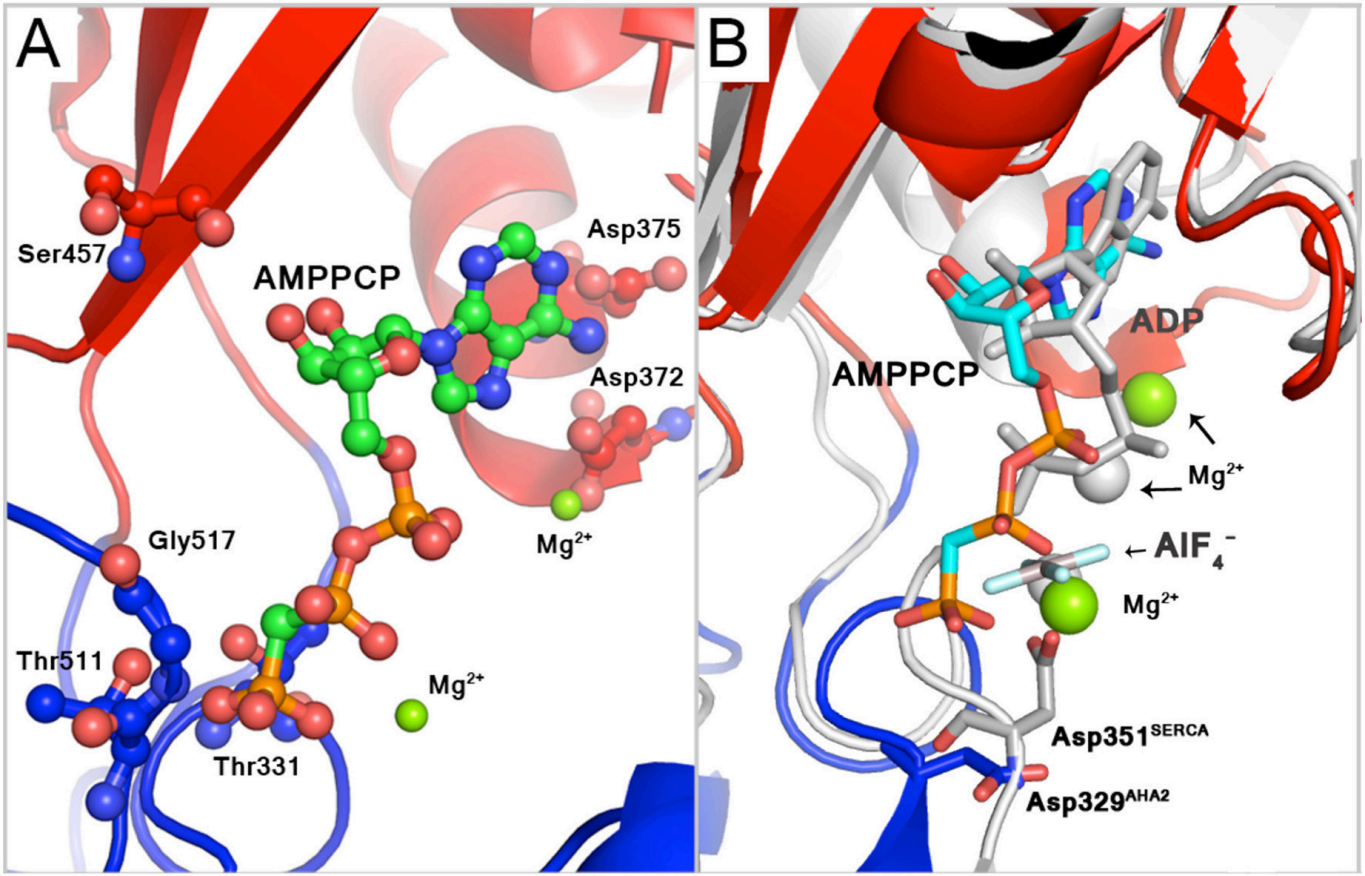
**Binding of AMPPCP. (A)** Binding of the phosphate groups of AMPPCP includes two Mg^2+^ ion, side chains of Thr511 and Thr331 and backbone atoms of Gly512 and Thr331. The adenine base can interact with side chains of Asp375 and Asp372, the latter of which also interacts with the Mg^2+^ ion that coordinates α-phosphate group. The ribose ring interacts with the backbone carbonyl of Ser457. **(B)** AHA2 binding of AMPPCP (green carbons) compared to ${\text{ADP}-\text{AlF}}_{4}^{-}$ (gray) binding to SERCA in the E1P-like state, pdb id: 1T5T (Sørensen et al., 2004). Mg^2+^—green spheres in AHA2, gray spheres in SERCA.

Alignment of the AHA2 and SERCA E1 structures (Figure 1A) shows that the cytoplasmic domains of AHA2 adopt a more compact conformation around the bound AMPPCP. Details of the coordination of the nucleotide are different than previously reported for AHA2. The adenine base of AMPPCP is rotated by ~60° resulting in suitable conformation for interactions with the N-domain via residues Asp372 and Asp375, while Ser457 interacts with the ribose (Figure 4) of AMPPCP. The γ-phosphate of AMPPCP is placed ~5 Å from Asp329 and coordinated only by P domain residues (Thr331, Thr511, Gly512) and the nearby Mg^2+^ ion, which also interacts with the β-phosphate. A second Mg^2+^ coordinates Asp372 of the N-domain.

**Figure 5:**
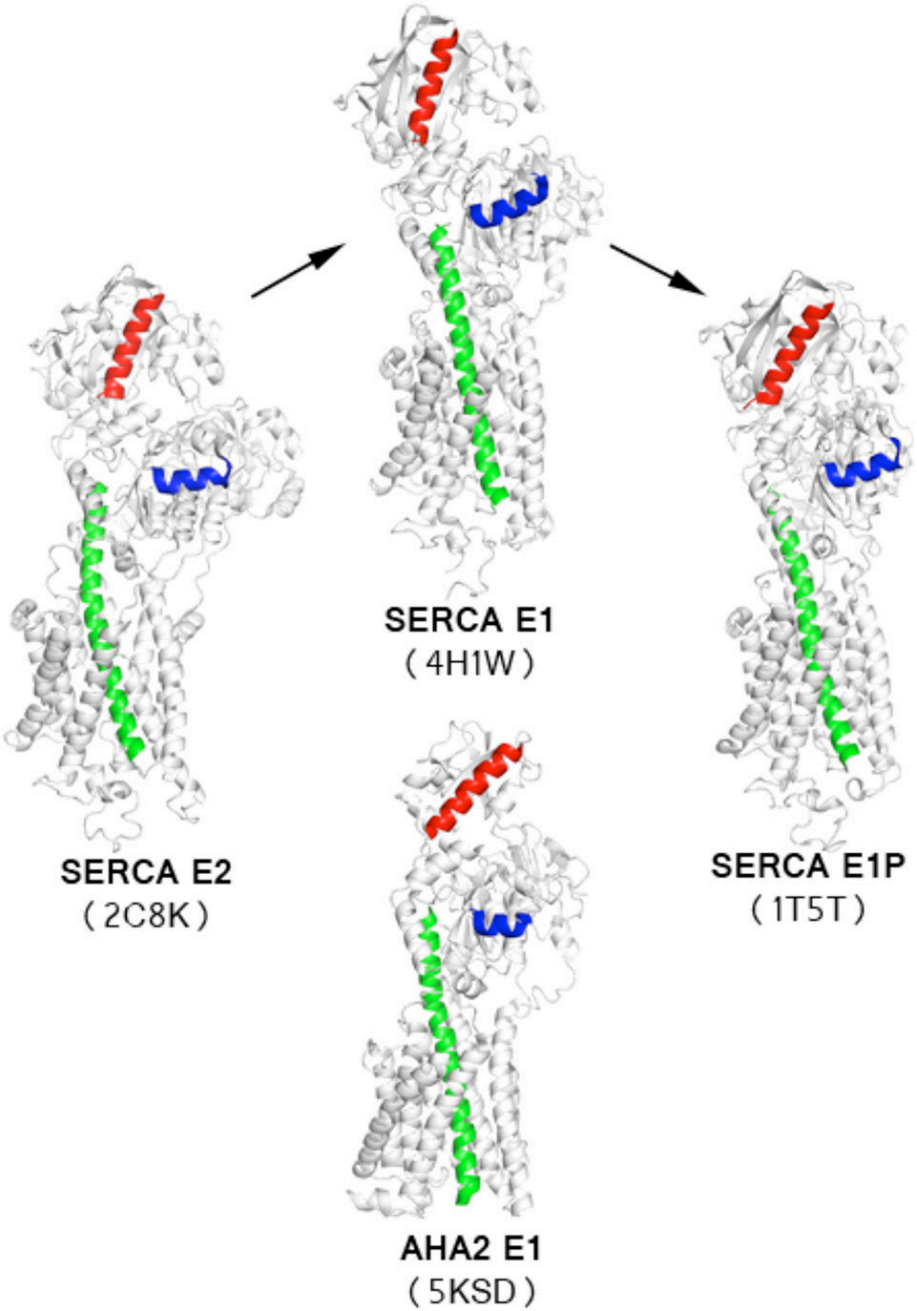
**Comparison of AHA2-AMPPCP model with available SERCA structures**. Orientation of a reference helix from the P-domain (blue) shows that the AHA2-E1 structure adopts an intermediate state of the transition between SERCA-E2 and SERCA-E1 states. Structures were aligned using helices TM5-TM9. TM5 marked in green. Corresponding helices from the N-domain are marked in red.

**Figure 6:**
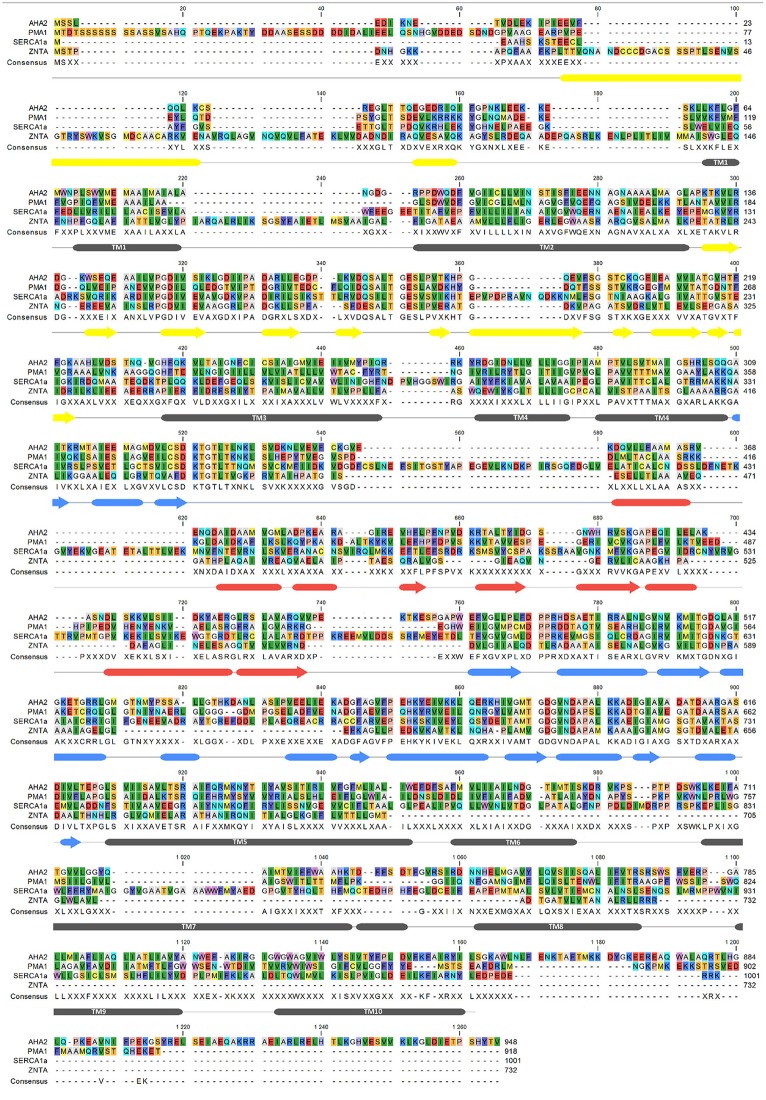
**Sequence alignment of AHA2, SERCA, PMA1, ZntA**. Secondary structure markings (below the aligned sequences) are based on AHA2 crystal structure with tubes for α-helix, arrows–β-strand, and lines for coil. The alignment was performed with MUSCLE (Edgar, 2004), using CLC Workbench (Genomics Workbench 7.7).^1^ and accession numbers: AHA2–P19456; PMA1–P05030; SERCA1a–P04191; ZntA–Q3YW59.

**Figure 7:**
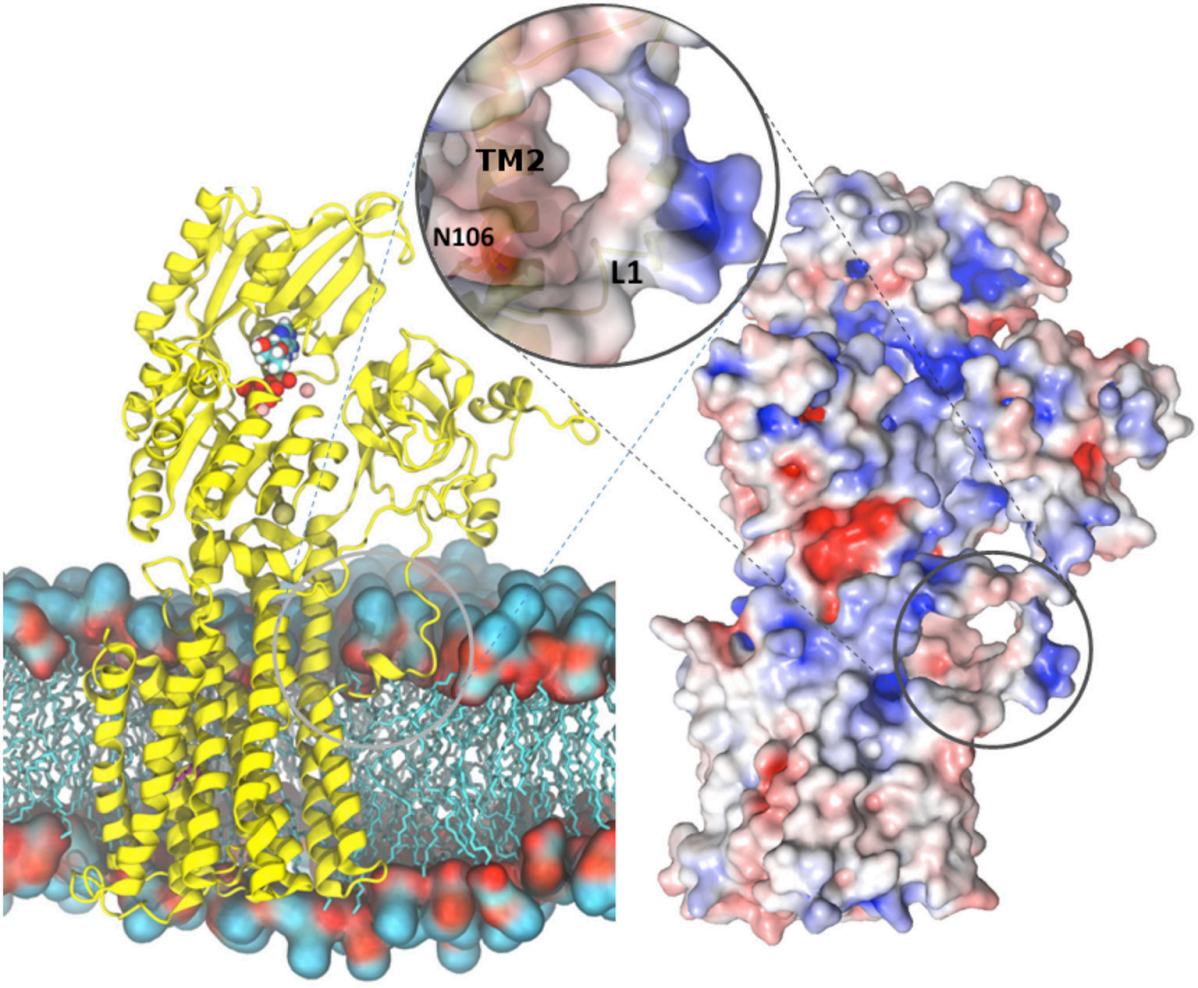
**Overview of the AHA2-AMPPCP structure. (Left)** AHA2 (yellow cartoon) modeled into a lipid bilayer. Ions and AMPPCP are shown by sphere representation. The circle zooms onto a negatively charged pocket formed between TM1 and TM2 in a close proximity of the Asn106-Asp684 pair. **(Right)** Overall surface electrostatic potential of AHA2 ± 5 K_b_T/e_c_ indicated in blue (positive) and red (negative) mapped on a surface representation. Negative electrostatic charge comes from two Glu residues situated in helix 2. The unstructured loop connecting the A domain and TM1 carries positive charges (blue color), which may interact with lipid head-groups.

Ion binding and occlusion at the membraneous site accompanies the formation of the catalytically competent E1P-like state (Olesen et al., 2007; Toyoshima et al., 2013; Winther et al., 2013). The transmembrane (TM) region however do not overlap when the two structures are aligned by their P-domains indicating that the SERCA and AHA2 E1 structures may have been captured at different intermediates of the E2 to E1P trajectory (Figure 5). The TM1 helix (see alignment in Figure 6) is differently placed, but shows both for AHA2 and SERCA an amphipathic association with the membrane interface (Figure 7). The TM segments 2–10 align with an r.m.s.d of 3.07 Å for Cα atoms. The K^+^ binding site was previously visualized using AHA2 E1-AMPPCP cocrystallized with rubidium chloride (Ekberg et al., 2010b). The re-refined AHA2 structure revealed this site directly at the P domain, where the K^+^ is coordinated by back bone oxygens of Lys597, Ala599, Asp600, Ala615 and by side chain oxygens of highly conserved Asp617 (Figure 2).

### Proton binding site at the conserved Asp684

**Figure 8:**
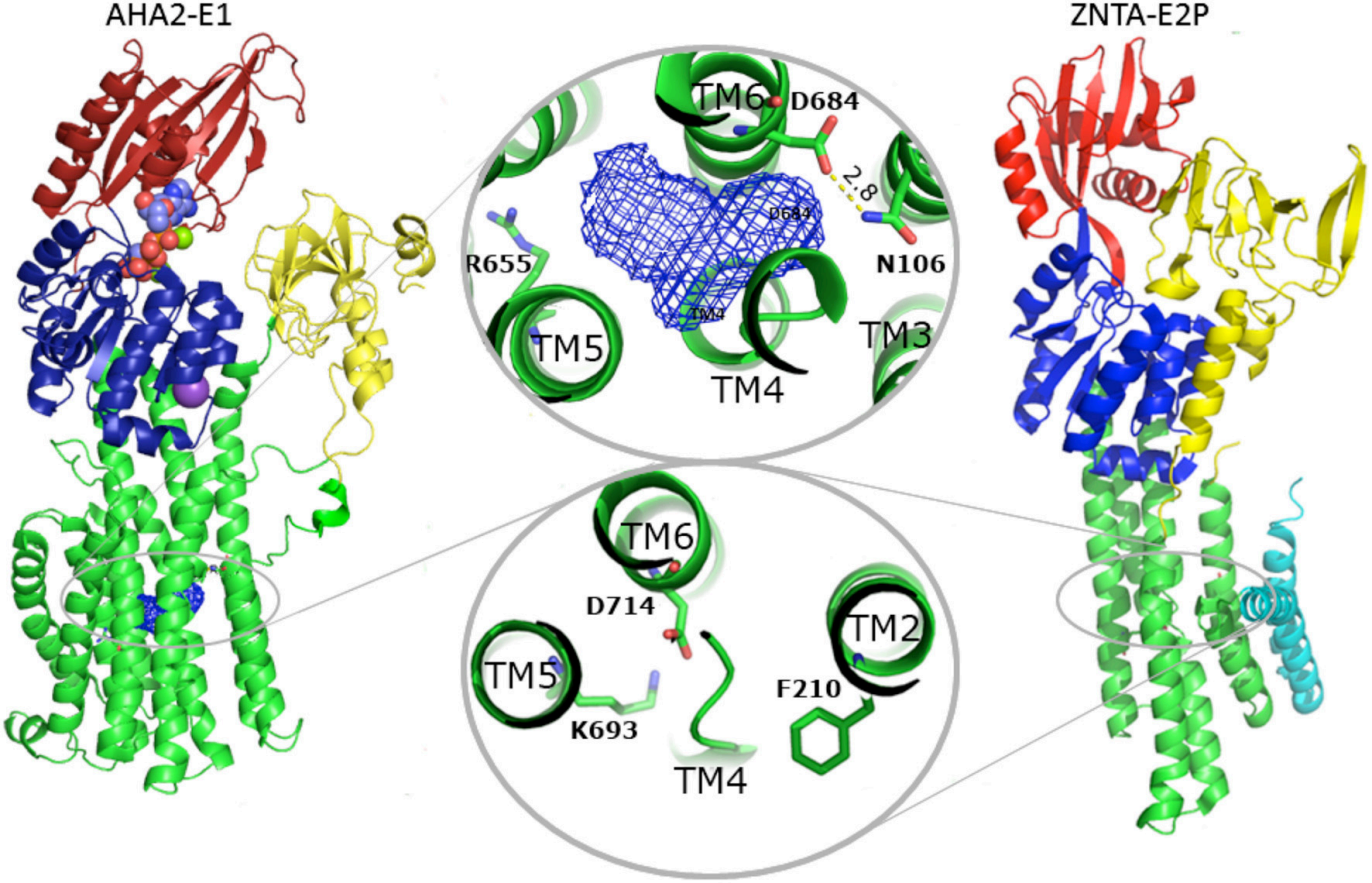
**Comparison between AHA2 and Zn^**2+**^-ATPase**. Despite different overall structure, the TM domains of AHA2 and the *Shigella sonnei* Zn^2+^-ATPase ZntA exhibit significant resemblance regarding the distribution of charged residues in the TM helices associated with transport. In both cases the TM helix 2 harbors a residue involved in closure of the ligand pathway from the cytoplasmic site. Phe210^ZntA^serves as a gating residue, while overlapping Asn106^AHA2^ is implicated in stabilization of the occluded, protonated Asp684. The Asp side chains placed in both cases in TM6 (Asp684^AHA2^ and Asp714^ZntA^) likely fulfill similar roles. Zinc binding in ZntA most likely involves Asp714 (and two conserved cysteines of TM4, not shown), while zinc release and the phosphorylation is stabilized by interactions with the conserved Lys693 of TM5. In AHA2 the equivalent Asp684 is essential for transport and most likely becomes deprotonated by interaction with Arg655 of TM5 along with E2P dephosphorylation.

Concerning proton transport and yeast PMA knock-out complementation, Asp684 of AHA2 is an indispensable, titratable residue in the TM domain (Buch-Pedersen et al., 2000). It is therefore considered the central binding site in proton transport. The superpositioning of the TM domains of AHA2 and SERCA shows a remarkable overlap of Asp684 and the critical Asp800 of SERCA that coordinates Ca^2+^ at both sites I and II in SERCA (Toyoshima et al., 2000). A cavity in AHA2 localized between TM helices 4, 5, and 6 connects Asp684, which is paired to Asn106 of TM2 in the current structure, and the Arg655 residue of TM 5 (Figure 8, and see below).

Some Asp684 mutants are still functional ATPases, but show no proton transport, except for the D684E mutation that also complements a yeast PMA knock-out. Remarkably, the Asp684 mutants were more than 1,000-fold less sensitive to vanadate, suggesting that they accumulate in the E1P state (Buch-Pedersen and Palmgren, 2003) as also supported by proteolytic cleavage analysis (Buch-Pedersen et al., 2000). In other words, an occluded structure is likely reachable to support phosphorylation of Asp684 mutated forms, but no proton release mechanism will subsequently stimulate turn-over of the phosphoenzyme that therefore accumulates in E1P.

### Proton occlusion at the Asp684-Asn106 pair

**Figure 9:**
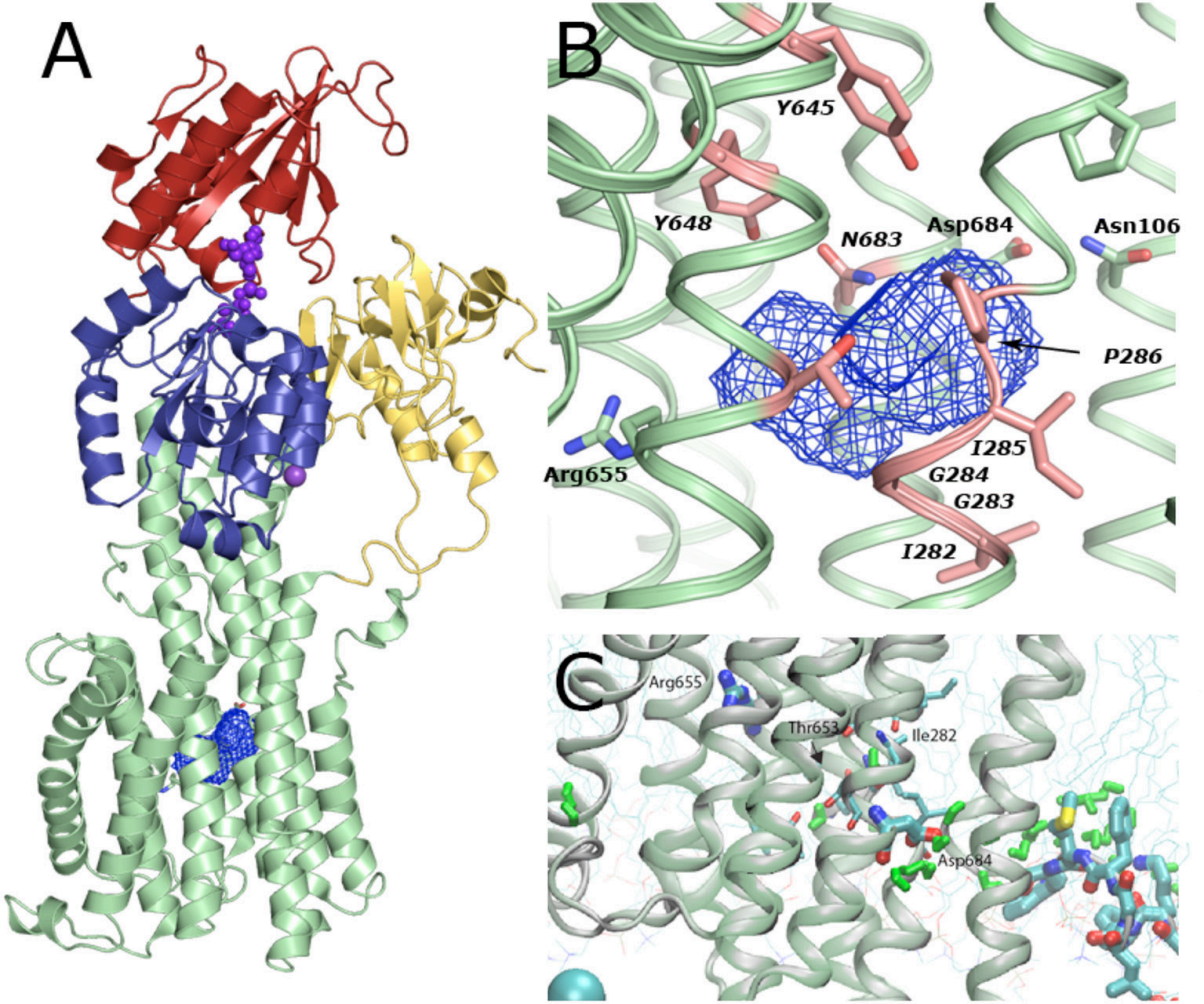
**Solvent cavity in the TM domain of AHA2. (A)** Overview on the cavity location within the TM domain. The cavity likely encloses 8–10 bound water molecules in this conformation of the pump. **(B)** Zoom-in of the cavity viewed in the plane of the membrane. The cavity is formed by backbone atoms of Ile282, Gly283, and Ile285 along with Gly 284, Pro286 and side chains of conserved Tyr645, Tyr648, Tyr653, and Arg655 of TM5 and Asn683 of TM6. **(C)** Molecular Dynamics simulations indicate that a continuous string of water molecules (shown in green) can reach from the cytoplasm to the Asp684 residue, the proposed proton binding site, i.e., an open proton translocation pathway, which must close later in the functional cycle, most likely along with E1P formation. Two stable water molecules reached almost half way through the membrane into the TM domain where they can interact with Ile282, Thr653 and backbone oxygen of Ala649, and Cys247, next to the central solvent cavity.

An important role of Asn106 was apparent from the structure of AHA2 (Pedersen et al., 2007). It is conserved in all P_III_-type H^+^-ATPases and positioned close to a large intramembraneous cavity (Figure 9), where it pairs with Asp684. The proximity of the two residues is compatible with formation of a neutral hydrogen-bonded pair between a protonated Asp684 and Asn106 as a basis for a stable proton binding site associated with the occluded E1P state (Figure 8). Similarly, occlusion of the Asp684-Asn106 pair is likely to increase the pKa of Asp684 and therefore stabilize protonation (Buch-Pedersen et al., 2009). The important functional role of Asn106 was further highlighted by mutational studies (Ekberg et al., 2013). Various point mutations (N106A, N106D, N106K, N106Q, and N106T) could complement a PMA1 knockout and maintain a membrane potential. Kinetic characterization of the purified mutant proteins confirmed their ability to hydrolyze ATP and transport protons, however at reduced rates as compared to the WT pump and with an acidic shift in the pH dependence as indeed to be expected from reduced stabilization of the protonated Asp684. Notably, a three-fold increased vanadate sensitivity of the N106D mutant suggested that relative to the wildtype it is shifted toward an outward-oriented E2 state (Ekberg et al., 2013).

## Proton entry pathway

Associated with E1 function, a proposed cytoplasmic proton entry pathway is located at the N-terminal part of the membrane domain at the cytoplasmic interface. A smaller cavity is evident above the ^TM2^Asn106-^TM6^Asp684 pair, and is defined by residues of helices TM2, 4, 5, 6, and 8, including two negatively charged glutamates (Glu113 and 114 of TM2) that may both attract protons and repel negatively charged lipids from blocking the cavity. MD simulations based on the improved model suggest a “U”-shaped solvent tunnel between TM1, 2, 4, and 6 and support the earlier proposal (Pedersen et al., 2007) that solvent enters the cavity from the cytoplasmic environment and thereby provides transmission of a proton to the Asp684-Asn106 pair.

The N-terminal end of TM1 is in close proximity to the cavity and the proposed solvent tunnel. TM1 is significantly shorter compared to the other helices with Pro68 (conserved among P_III_-type members; Axelsen and Palmgren, 1998) defining the N-terminal starting point. The preceding A/TM1 linker is poorly defined in the crystal structure. Two positively charged lysine residues (Lys57 and Lys60) may interact with head-groups of the phospholipid membrane. Indeed, MD simulations show this region to partition into the membrane interface through an amphipathic helix for residues 56–64 and to induce a local depression in the membrane that may facilitate the solvent access to the Asp684-Asn106 pair in the current E1 state (Figure 9). A similarly short and kinked TM1 helix is also seen in SERCA, and for the SLN bound E1 structure of SERCA it is as well immersed into the membrane and may facilitate Ca^2+^ entry, described as a “sliding door mechanism” (Winther et al., 2013). A similar TM1 structure is observed for Na^+^,K^+^-ATPase (Morth et al., 2007) and a role in Na^+^ entry proposed (Einholm et al., 2007; Laursen et al., 2009). The heavy-metal transporting P_1B_-ATPases show a different N-terminal topology with a heavy-metal binding domain and two additional N-terminal transmembrane segments MA and MB, but here again the MB helix is short and kinked and has been implicated in the mechanism of heavy metal entry for the membraneous site (Gourdon et al., 2011; Wang et al., 2014). A kinked (near-) N-terminal helix distorting the cytoplasmic membrane interface therefore appears as a general mediator of substrate entry in P-type ATPases.

### Proton transport pathway through the membraneous cavity

Proton release is connected to the E2P state, which is generally characterized by low ATP and E1 substrate affinity. The proposed transport mechanism with the Asp684 side chain functioning as a titratable proton acceptor/donor site raises the question of how protons are then extruded in an outward-open E2P form? Next to Asp684, toward the extracellular side, we observe a large intramembranous cavity of ~265 Å^3^ formed between helices TM4–TM6. This cavity is mostly due to the unwound structure of the TM4 helix at conserved Pro286 and Pro290 residues (Bukrinsky et al., 2001), and a bulged structure at the Asp684 position contributes as well. A similar deformation of the TM4 helix is present in e.g., SERCA, Na^+^,K^+^-ATPase, CopA, and ZntA, yet in a very different context of metal cation coordination. Backbone carbonyl and amide groups from AHA2 residues Ile282, Gly283 and Ile285 along with Gly284, Pro286 and side chains of conserved Tyr645, Tyr648, Tyr653, and Arg655 of TM5 and Asn683 of TM6 define the cavity surface, which therefore appears surprisingly polar. Ile282 has been implicated in proton translocation (Fraysse et al., 2005). Our MD simulations further suggest a role of Ile282 and Tyr653 in allowing water molecules to penetrate the cavity (Figure 9).

Homology modeling of the E2P state suggested an important role for Arg655 in TM helix 5 approaching toward the Asp684 and Asn106 pair to stimulate deprotonation. Also the E2P modeling suggests that the water-filled cavity merges into a then deep, solvated extrusion pathway leading from Asp684 and pass Arg655 to the extracellular environment (Figure 8). Arg655 is conserved among all plant PM H^+^-ATPases and corresponds to a His residue of similar functionality in fungi (see below). The equivalent residue in SERCA is the Ca^2+^ binding Glu771 (Figure 6), which indeed faces the extracellular pathway in the E2P state. The exact conformation and interactions of the positively charged Arg655 side chain in AHA2 cannot be reliably defined at the current resolution, albeit its proximity effect on Asp684 deprotonation and its interaction with the water-filled cavity appears quite likely and as a simple model of function.

The role of Arg655 in the catalytic cycle of AHA2 has been investigated by extensive mutagenesis and functional assays (Buch-Pedersen and Palmgren, 2003). Out of three prepared mutants (R655K, R655A, R655D) the R655K mutant was reported to support growth at a WT level in a PMA1 knockout yeast strain, while R655A and R655D could not complement at all. ATP affinity was unaltered for all mutant (confirming proper folding and preserved functionality of the N-domain), and the pH-dependence of the enzyme was maintained indicating preserved function of proton binding. However, the hydrolytic activities of the mutant pumps were significantly reduced. Vanadate insensitivity and enzyme phosphorylation levels indicated an accumulation in the E1P state (Buch-Pedersen and Palmgren, 2003) in full agreement with a proposed role in proton release. Double mutants of the Asp684 and Arg655 sites showed that only R655K/D684E was able, to some extent, to sustain yeast growth in a PMA knockout.

Importantly, a similar mechanism has been proposed for the bacterial Zn^2+^-ATPase where Zn^2+^ release in the E2P state could be facilitated by the positively charged K693 residue, which indeed stabilizes the Zn^2+^ binding Asp714 as a built-in counter ion (Wang et al., 2014) in the Zn^2+^-free E2 Pi state (Figure 8). Localization of this lysine at the exit of the ion pathway suggests that it too can block re-entry, in this case of extracellular Zn^2+^ ions to the transmembrane domain (Wang et al., 2014). Functional experiments have failed to identify any counter-transported ligand for the fungal and plant proton pumps supporting the model of Arg655 as a built-in counterion (Pedersen et al., 2007). In the fungal proton pump, which sustains even stronger polarization of the membrane potential than the plant protein (Blatt et al., 1987), the position corresponding to the Arg655 is occupied by His701 (yeast PMA1 numbering) and in close proximity to Arg649 located next to the water-filled cavity. Such clustered arrangement of positively charged residues at the proton release pathway may explain the remarkably steep membrane potentials that can be attained in fungal cells through PMA activity.

### Comparison to other proton transporters

The functional role of Arg655 and Asp684 in the AHA2 proton transport mechanism is similar in concept to many other proton transport proteins such as bacteriorhodopsin (Pebay-Peyroula et al., 1997; Luecke et al., 1998) and F-/V-type ATPases (Hutcheon et al., 2001; Fillingame and Dmitriev, 2002) where an Arg-dependent pKa shift of a carboxylic acid side chain facilitates the proton transfer (Buch-Pedersen et al., 2009). Localization of Arg655 at the exit pathway of the proposed proton transport channel suggests that it can also function as a built-in counter ion that facilitates E2P occlusion and dephosphorylation and as a positively charged block for extracellular proton re-entry (Pedersen et al., 2007). The proposed behavior of the Asp684 and Arg655 residues in AHA2 resembles mechanisms utilized by other proton transporters. In case of F_1_F_0_-ATPase, the proposed gating of periplasmic H^+^ is based on periodic formation of a salt bridge at the interface of the transmembrane *a* subunit and a rotor-like *c* subunit. The relative position of the involved residues, *a*Arg210 and *c*Asp61, promotes either a formation of the salt bridge which facilitates a proton release to the cytoplasmic half-channel of the ATP synthase, or, when the salt bridge is broken due to movement of the *a* subunit triggered by acidification, allow for protonation of an available *c*Asp61 residue (Dong and Fillingame, 2010).

A mechanism similar to the function of the Asn106-Asp684 pair observed in AHA2 was proposed for the *Escherichia coli* ClCec Cl^−^/H^+^ antiporter and cytochrome C oxidase. Based on a crystal structure of the former protein, a Glu148 residue, located at the beginning of the translocation pathway, was suggested to serve as a gate for the ions, changing its side chain conformation in response to e.g., pH (Dutzler et al., 2003; Accardi and Miller, 2004). In cytochrome c oxidase a proton pathway has a form of a ~25 Å long cavity, made by polar residues and several ordered water molecules. From the crystal structures it was noticed that an Asn139 residue affects formation the integrity of a water chain that supports proton translocation (Iwata et al., 1995; Tsukihara et al., 1996; Svensson-Ek et al., 2002; Qin et al., 2006). Free-energy simulations visualized a metastable rotamer state where the residue changes the side chain conformation and opens the channel to form a functional ion translocation pathway (Henry et al., 2009).

## Conclusions

The application of iMDFF environment in refinement of low-resolution protein structures was successfully reported in studies on Human Insulin Receptor Ectodomain (Croll et al., 2016), and we have applied it here to the original, highly anisotropic 3.6 Å resolution crystallographic data obtained from AHA2 crystals (Pedersen et al., 2007). Structural changes of the revised AHA2 model includes a local rearrangement of transmembrane helices 7 and 8, where ~1-turn N-terminal register shift is observed, and local changes at the nucleotide binding pocket of the N-domain. Additionally, improvement in the model quality allowed for inclusion of a conserved K^+^-binding site located at the P-domain.

The improved quality of the model provides a more confident basis of the proposed H^+^ transport mechanism utilized by P_III_-type ATPases. The proton translocation pathway, which centers on earlier identified residues Asn106, Asp684, and Arg655, begins at the cytoplasmic side of the TM domain from where protons are delivered to the Asn-Asp pair via a solvent tunnel located between TM1, 2, 4, and 6. Solvent accessibility of the proton entrance is obtained by a characteristically short, helical structure of TM1. Furthermore, the angle between the P-domain and the membrane maybe important for the function of this entry pathway. Based on modeling of E1P-E2P conformational changes, protons are likely transported via a large solvent-filled cavity that merges with an exit pathway toward the extracellular side of the membrane. Arg655, proximal to the cavity stimulates deprotonation of Asp684 and proton release, serving as a built-in counter ion required for E2P closure and dephosphorylation and the E2-E1 transition of the pump.

The AHA2 structure reveals mechanistic concepts that can also be recognized in other transmembrane proton/cation transport systems, yet future structures of E2 states will be required to fully elucidate functional transitions of P_III_-type H^+^-ATPases. Interestingly, single-molecule studies of AHA2 function have been introduced and suggest also that inactive states and protein-mediated proton leaks must be considered as part of the functional cycle (Veshaguri et al., 2016), thus many structural and mechanistic questions remain to be addressed to get a full insight into the inner workings of the plasma-membrane proton pump.

## Author note

We dedicate this paper to the memory of the late Carolyn Slayman, a pioneer of the plasma-membrane proton pump field.

## Endnote

The re-refined coordinates are available in the Protein Data Bank through accession number 5KSD.

## Author contributions

TC performed new refinement, assisted by BP and PN. DF drafted the paper and performed structural analysis. BP and PN supervised the project.

## Funding

TC was supported by a visiting scientist fellowship by the Aarhus University Research Foundation (AUFF). BP was supported by an Marie Curie Co-fund fellowship from the Aarhus Institute for Advanced Studies (AIAS) and by a Sapere Aude fellowship from the Danish Council for Independent Research (DFF-FNU). The work was supported by the PUMPkin Centre of excellence of the Danish National Research Foundation, and the DANDRITE center co-financed by Lundbeckfonden and Aarhus University.

### Conflict of interest statement

The authors declare that the research was conducted in the absence of any commercial or financial relationships that could be construed as a potential conflict of interest.

## Footnotes

^1^https://www.qiagenbioinformatics.com/
